# Tryptophan catabolism and immune activation in primary and chronic HIV infection

**DOI:** 10.1186/s12879-017-2456-z

**Published:** 2017-05-16

**Authors:** Marco Gelpi, Hans J. Hartling, Per M. Ueland, Henrik Ullum, Marius Trøseid, Susanne D. Nielsen

**Affiliations:** 1 Viro-Immunology Research Unit, Department of Infectious Diseases, University Hospital of Copenhagen, Rigshospitalet, Blegdamsvej 9, Copenhagen, Denmark; 2 Section for pharmacology, Department of Clinical Science, University of Bergen, Bergen, Norway; 3 Department of Clinical Immunology, University Hospital of Copenhagen, Rigshospitalet, Blegdamsvej 9, Copenhagen, Denmark; 4 Section of Clinical Immunology and Infectious Diseases, University Hospital Rikshospitalet, Kirkeveien 166, Oslo, Norway

**Keywords:** HIV, Kynurenine/Tryptophan ratio, Primary HIV infection, Immune activation

## Abstract

**Background:**

Kynurenine/Tryptophan ratio (KTR) is increased in HIV infection, and linked to immune activation. We hypothesized that early cART initiation results in lower KTR compared to late initiation. Furthermore, we hypothesized that KTR prior to cART is a predictor of the magnitude of subsequent reduction in immune activation.

**Methods:**

Prospective study including 57 HIV-infected individuals (primary HIV infection (*N* = 14), early presenters (>350 CD4+ T cells/μL, *N* = 24), late presenters (<200 CD4+ T cells/μL, *N* = 19)). Kynurenine and tryptophan were analysed by liquid chromatography–tandem mass spectrometry. Total CD4+ and CD8+ T cells were determined and proportion of activated CD38 + HLA-DR+ Tcells was measured using flow cytometry at baseline and after 6 and 12 months of cART.

**Results:**

At baseline, primary HIV infection had higher KTR than early presenters. However, similar KTR in primary HIV infection and early presenters was found after cART initiation, while late presenters had higher KTR at all time points. In primary HIV infection and early presenters, KTR was positively associated with proportion of activated cells at baseline. Furthermore, in early presenters the KTR at baseline was associated with proportion of activated cells after 6 and 12 months. Interestingly, in primary HIV infection the KTR at baseline was positively associated with reduction in proportion of CD8 + CD38 + HLA-DR T cells after 6 and 12 months.

**Conclusions:**

Lower kynurenine/tryptophan ratio during follow-up was found after early initiation of cART. KTR in primary HIV infection and early presenters was positively associated with immune activation. Importantly, KTR in primary HIV infection predicted the magnitude of subsequent reduction in immune activation. Thus, a beneficial effect of early cART on KTR was suggested.

## Background

Primary HIV infection is characterized by rapid depletion of CD4+ T-cells, high viral load, and the establishment of a chronic state of immune activation [1]. The magnitude of these three events has been shown to be closely related to the long term prognosis in HIV-infected individuals [2, 3]. Thus, higher CD4+ T-cell counts and lower levels of chronic immune activation are associated with a better clinical outcome [4, 5].

Tryptophan is an essential amino-acid, metabolized by the kynurenine pathway. Rate-limiting factors in this pathway are Tryptophan 2,3-dioxygenase (TDO) and Indoleamine 2,3-dioxygenase (IDO). TDO and IDO are active mainly in physiological conditions and conditions characterized by immune activation, respectively [6, 7]. Furthermore, minor amounts of IDO-like enzymes are produced by gut bacteria [8]. This activity has been described to be enhanced in HIV-infected individuals [8, 9].IDO catalyzes the catabolism of tryptophan to N-formylkynyrenine, which is rapidly decomposed to kynurenine [10, 11]. Thus, the ratio between kynurenine and tryptophan (KTR) in plasma can be used as an indirect measure of IDO activity [12]. Increased KTR has been described in numerous conditions characterized by low grade inflammation, such as Alzheimer’s disease, cancer, and viral infections [13, 14]. We and other groups have described an increased KTR in HIV infection [6, 9, 15–19]. Tryptophan catabolites, in particular kynurenine and quinolinic acid, are associated with immune activation [20, 21], and increased KTR is associated with a higher level of immune activation in untreated HIV-infected individuals [15, 22]. Furthermore, tryptophan catabolism has been linked to CD4+ immune regulation. In particular, an inverse association between KTR and the ratio between Th17 cells and T regulatory cells in both primary and chronic phases of HIV infection has been described [12, 23].

An early initiation of combination antiretroviral therapy (cART) is associated with both a lower level of immune activation and faster CD4+ T-cell recovery compared to initiation of treatment in later stages of HIV infection [24, 25]. A reduction of KTR following cART initiation has been described by several recent studies [6, 15, 17–19]. However, it is unclear whether early initiation of cART results in a greater reduction or even normalization of the KTR set point compared to cART initiation in late presenting HIV infection.

In the present study, we hypothesized that early initiation of cART during primary HIV infection or in early presenting patients with CD4+ T cell counts above 350 cells/μL results in improved KTR set point compared to initiation of cART in late presenters with CD4 T cell counts below 200 cells/μL. Furthermore, we hypothesized that KTR before initiation of cART is a predictor of both CD4 T-cell recovery and the magnitude of the subsequent reduction in immune activation. To investigate this, KTR was measured before initiation of cART and after 6 and 12 months of cART in individuals with either primary HIV infection or chronic HIV infection. To our knowledge, this is the first prospective study of KTR in a cohort including both individuals with primary and chronic HIV infection.

## Methods

### Study population

A total of 100 HIV-infected individuals that initiated cART were included in a prospective cohort study [26], and plasma samples were obtained at baseline, and at 6, 12, and 24 months after initiation of cART as previously described [26]. Inclusion criteria were a positive HIV-1 test and initiation of cART. Decision about cART initiation was made by the patient’s physician and the patient, and participation in this study did not influence this decision. All individuals were enrolled from Department of Infectious Diseases, Rigshospitalet, Copenhagen University Hospital or Department of Infectious Diseases, Hvidovre Hospital. In the present study, 57 treatment naïve HIV-infected individuals were included. We included all patients from the cohort belonging to either of the three groups: primary HIV infection (*N* = 14), early presenters (>350 CD4+ T cells/μL at baseline, *N* = 24), or late presenters (<200 CD4+ T cells/μL at baseline, *N* = 19) and with plasma samples available both at baseline and at least at one of time points 6 and 12 months of follow-up. Only few samples were available at 24 months of follow-up, these samples were therefore not included in the present study. A total of 14 individuals were defined as having primary HIV infection, i.e. they had a negative HIV test less than 6 months prior to diagnosis and/or one or more negative bands in Western Blot. Fiebig stages for individuals with primary HIV infection were as follows: I, *N* = 1; II, *N* = 1; III, *N* = 1; IV, *N* = 11. Furthermore, 16 healthy individuals were included as healthy controls. No differences in age and sex distributions were found between the groups (Table 1).

**Table 1:** Clinical characteristics of the study population

	Primary HIV infection *N* = 14	Early presenters *N* = 24	Late presenters *N* = 19	Healthy controls *N* = 16	*P*
Gender, males/females, (% males)	13/1 (92.9)	22/2 (91.7)	18/1 (94.7)	15/1 (93.8)	.948
Age, years, mean (SD)	46 (8.9)	45 (8.2)	47 (10.1)	43 (9.4)	.529
CD8+ at baseline, cells/μL, mean (SD)	1238.6 (659.8)	1123.5 (547.5)	820.1 (556.0)	915.7 (409.7)	.116
CD4+ at baseline, cells/μL, mean (SD)	577.8 (228.5) ^a,d^	527.9 (111.2)^c,f^	72.7 (62.7)^a,c,e^	1144.0 (329.0)^d,e,f^	< .001
CD4/CD8 at baseline, mean (SD)	0.6 (0.4)^a,d^	0.6 (0.2)^c,f^	0.1 (0.1)^a,c,e^	1.4 (0.5)^d,e,f^	< .001
Co-infection with chronic HBV/HCV, N	0/1	1/0	0/2	0/0	NA
HIV RNA at baseline, mean (SD)	2,413,322 (4,119,479)^a,b^	97,328 (253,967)^b,c^	486,483 (524,072)^a,c^	NA	< .001
AIDS defining events, N	0	0	1	NA	NA
KTratio at baseline, mean (SD)	46.8 (20.3)^a,b,d^	34.8 (10.6)^b,c,f^	74.5 (48.1)^a,c,e^	20.9 (3.7)^d,e,f^	< .001
CD8 + CD38 + HLA-DR+, % cells, mean (SD)	44.7 (20.3) ^a,b,d^	22.0 (13.4)^b,c,f^	25.5 (21.3)^a,c,e^	1.3 (0.8)^d,e,f^	< .001
CD4 + CD38 + HLA-DR+, % cells, mean (SD)	5.5 (4.1)^a,d^	3.5 (3.0)^c,f^	15.5 (15.4)^a,c,e^	0.8 (0.6)^d,e,f^	< .001

P*: comparing the four groups by using ANOVA if parametric variables or Kruksal-Wallis test if non parametric variables. If significant (<0.05) then t-test if parametric variables or Mann-Whitney if non parametric variables were used to compare two groups. Only significant differences are marked:

^a^primary HIV infection vs late presenters

^b^primary HIV infection vs early presenters

^c^late presenters vs early presenters

^d^healthy controls vs primary HIV infection

^e^healthy controls vs late presenters

^f^healthy controls vs early presenters

Written informed consent was obtained from all participants, and the study was performed in accordance with the ethical guidelines of the 1975 Declaration of Helsinki and approved by the Local Ethical Committee (H-3-2011-089) and the Danish Data Protection Agency.

### Collection of blood samples and flow cytometry

Blood samples from HIV-infected individuals were collected at the day of inclusion (baseline/day 0 of cART) and after 6 and 12 months of cART. Blood samples from healthy controls were only collected once at the time of inclusion. The numbers of samples available at each timepoint (baseline, 6, and 12 months after initiation of cART) were: primary HIV infection: 14, 11, 7; early presenters: 24, 22, 8; late presenters: 19, 10, 8.

Blood collected in ethylenediamine tetraacetic acid (EDTA) tubes was used for flow cytometry as described previously [26]. In brief, 100 μL of EDTA blood was incubated with fluorescent dye–conjugated monoclonal antibodies, erythrocytes were lysed with 2 mL of Lysing Solution (Becton Dickinson (BD), Franklin Lakes, NJ, USA), and the samples were washed and resuspended in Facs flow (BD). CD3 in combination with CD8 or CD4 was used to characterize T cells, and chronic activated CD4+ and CD8+ T cells were determined as proportion of CD4 + CD38 + HLA-DR+ and CD8 + CD38 + HLA-DR+ T cells, respectively. Acquisition was performed using a FACS Canto, and data were processed using FACS Diva software (BD). Monoclonal antibodies used to determine lymphocyte subsets were CD3 FITC, CD4 APC-Cy7-A, CD8- PerCP-Cy5–5-A, CD38 PE-Cy7-A, and HLA-DR APC-A and appropriate isotype controls all purchased from BD. For each sample a minimum of 100,000 cells were acquired and gated as previously described by our group [27] Lymphocyte subsets are given as the proportion (%) of the cell population concerned (CD4+ T cells or CD8+ T cells).

Furthermore, the reduction of immune activation after initiation of cART was determined as the difference between proportion of CD4+ or CD8+ T cells expressing CD38 + HLA-DR+ at baseline and after 6 and 12 months of follow-up (Δ % CD8 + CD38 + HLA-DR+ T cells = % CD8 + CD38 + HLA-DR+ T cells at one of the follow-up time points – % CD8 + CD38 + HLA-DR+ T cells at baseline).

### Kynurenine and tryptophan

Kynurenine and tryptophan were analysed at BEVITAL (www.bevital.no) by liquid chromatography–tandem mass spectrometry (LC-MS/MS) as previously described [28], and kynurenine/tryptophan ratio (KTR) was used as a measure of IDO-1 activation [12]. In the interest of clarity, the ratio is multiplied by 1000 and expressed as the KTR in this study.

### Statistical analysis

Parametric or non-parametric tests were used as appropriate. Differences between the groups of HIV-infected individuals were evaluated by one way ANOVA test (Kruksal-Wallis test in case of non-parametric distribution) and if significant followed by unpaired t tests or by Mann-Witney U tests in case of non-parametric distribution. The effect of baseline measures of KTR on immune recovery and immune activation after initiating cART were evaluated by Pearson’s and Spearman’s test, as appropriate. Two-tailed *P*-values <0.05 were considered significant. All statistical analyses were performed using SPSS (version 22 - IBM corp., Armonk, New York, USA).

## Results

### CD4+ and CD8+ T cell count and immune recovery

Clinical characteristics of the study populations are presented in Table 1.

At baseline, individuals with primary HIV infection and early presenters had comparable CD4+ T cell counts, while late presenters had lower CD4+ T cell counts than the two other groups (Table 1). Furthermore, at baseline all groups of HIV-infected individuals had lower CD4+ T cell counts than healthy controls (Table 1). No differences in CD8+ T cell count were found between groups. No differences in CD4+ T cell recovery (increase in CD4+ T cell count from baseline to a time of follow-up) were found between any of the groups of HIV-infected individuals at any time point. Results regarding immune recovery for the entire cohort have been presented in previous studies [26, 29].

### KTR in plasma

**Fig. 1:**
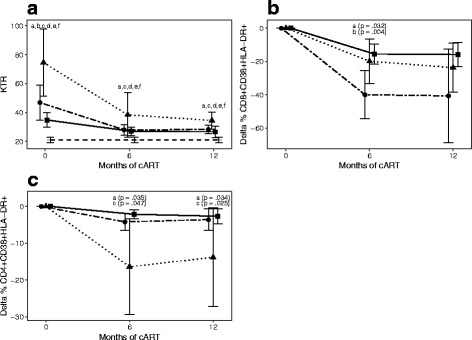
Kynurenine/Tryptophan ratio (KTR) in primary HIV infection, late presenters (LP) and early presenters (EP) (**A**). Reduction of proportion of CD8 + CD38 + HLA-DR+ (**B**) and CD4 + CD38 + HLA-DR+(**C**) T cells at 6 and 12 months of follow-up. The numbers of samples available at each timepoint (baseline, 6, and 12 months after initiation of cART) were: primary HIV infection (●): 14, 11, 7; late presenters (▲): 19, 10, 8; early presenters (■): 24, 22, 8; healthy controls (+): 16. P*: comparing the four groups by using ANOVA if parametric variables or Kruksal-Wallis test if non parametric variables. If significant (<0.05) then unpaired t-test if parametric variables or Mann-Whitney U test if non parametric variables were used to compare two groups. Only significant differences are marked: **a**: primary HIV infection vs late presenters; **b**: primary HIV infection vs early presenters; **c**: late presenters vs early presenters; **d**: healthy controls vs primary HIV infection; **e**: healthy controls vs late presenters; **f**: healthy controls vs early presenters

At baseline, KTR in individuals with primary HIV infection was higher than in early presenters and lower than in late presenters (Table 1). However, similar KTR in individuals with primary HIV infection and early presenters was found after 6 and 12 months of cART. In contrast, late presenters maintained higher KTR at 6 and 12 months of cART compared to both primary HIV infection and early presenters (Fig. 1a). Furthermore, all groups of HIV-infected individuals (primary HIV infection, early presenters, and late presenters) had higher KTR compared to healthy controls at all time points (Fig. 1a).

### Immune activation

At baseline, individuals with primary HIV infection had higher proportion of CD8 + CD38 + HLA-DR+ T cells compared to early presenters and late presenters. Furthermore, proportion of CD8 + CD38 + HLA-DR+ T cells at baseline was lower in early presenters than in late presenters (Table 1). Individuals with primary HIV infection and early presenters had similar proportion of CD4 + CD38 + HLA-DR+ T cells at baseline, both groups lower than late presenters (Table 1). After 6 and 12 months of cART, individuals with primary HIV infection, early presenters, and late presenters had comparable proportions of CD8 + CD38 + HLA-DR+ and CD4 + CD38 + HLA-DR+ T cells (Table 2). However, all groups of HIV-infected individuals had higher proportions of CD8 + CD38 + HLA-DR+ and CD4 + CD38 + HLA-DR+ T cells than healthy controls at all time points (Tables 1 and 2).

**Table 2:** Proportion of CD8 + CD38 + HLA-DR+ and CD4+ CD38 + HLA-DR+ T cell subsets in HIV-infected individuals with either primary HIV infection or chronic HIV infection before and after initiation of cART

		Primary HIV infection *N* = 14	Early presenters *N* = 24	Late presenters *N* = 19	Healthy Controls *N* = 16	*P*
% Cells	CD8 + CD38 + HLA-DR+					
	Baseline	44.7 (20.3)^a,b,d^	22.0 (13.4)^b,c,f^	25.6 (21.3)^a,c,e^	1.3 (0.8)^d,e,f^	<.001
	After 6 months of cART	6.4 (5.6)^d^	8.1 (5.5)^f^	6.5 (5.3)^e^	NA	<.001
	After 12 months of cART	8.9 (8.1)^d^	4.0 (1.9)^f^	4.7 (2.8)^e^	NA	<.001
	CD4 + CD38 + HLA-DR+					
	Baseline	5.5 (4.08)^a,d^	3.5 (3.0)^c,f^	15.5 (15.4)^a,c,e^	0.8 (0.6)^d,e,f^	<.001
	After 6 months of cART	1.6 (1.4)^d^	1.6 (1.3)^f^	3.3 (3.4)^e^	NA	<.001
	After 12 months of cART	1.6 (0.7)^d^	1.1 (0.3)	2.4 (1.7)^e^	NA	<.001

Data are shown as mean (SD)

P*: comparing the four groups by using ANOVA if parametric variables or Kruksal-Wallis test if non parametric variables. If significant (<0.05) then t-test if parametric variables or Mann-Whitney if non parametric variables were used to compare two groups. Only significant differences are marked:

^a^primary HIV infection vs late presenters

^b^primary HIV infection vs early presenters

^c^late presenters vs early presenters

^d^healthy controls vs primary HIV infection

^e^healthy controls vs late presenters

^f^healthy controls vs early presenters

After 6 months of cART, a larger reduction (delta %) in the proportion of CD8 + CD38 + HLA-DR+ T cells was found in individuals with primary HIV infection compared to early presenters and late presenters (Fig. 1b). In contrast, a larger reduction in the proportion of CD4 + CD38 + HLA-DR+ T cells was found in late presenters than in individuals with primary HIV infection and early presenters, both after 6 and 12 months of follow-up (Fig. 1c).

### KTR associated with reduction in immune activation, but not with immune recovery

In individuals with primary HIV infection and early presenters, baseline KTR was positively associated with proportion of CD8 + CD38 + HLA-DR+ T cells at baseline (ρ .867, *p* = 0.002 and ρ .544, *p* = 0.033, respectively), and in early presenters also at 6 months (ρ .670, *p* = 0.006, Table 3). Furthermore, baseline KTR in early presenters was associated with CD4 + CD38 + HLA-DR+ T cells at baseline (ρ .689, *p* = 0.003) and at 6 months (ρ .711, *p* = 0.003) and at 12 months of follow-up (ρ .679, *p* = 0.022) (Table 3). No significant associations between baseline KTR and immune activation were found in late presenters.

**Table 3:** Association between KTR before initiation of cART, immune activation and reduction in immune activation at baseline and at 6, and 12 months of follow-up

	Primary HIV infection *N* = 14		Early presenters *N* = 16		Late presenters *N* = 19	
	*ρ*	*pvalue*	*ρ*	*pvalue*	*ρ*	*pvalue*
CD8 + CD38 + HLA-DR+						
Baseline	.867	**.002**	.534	**.033**	.311	.260
After 6 months of cART	−.417	.265	.670	**.006**	.152	.676
After 12 months of cART	−.800	.104	.451	.164	.145	.670
CD4 + CD38 + HLA-DR+						
Baseline	.267	.488	.689	**.003**	.530	.051
After 6 months of cART	−.410	.273	.711	**.003**	.527	.117
After 12 months of cART	−.872	.054	.679	**.022**	.045	.894
ΔCD8 + CD38 + HLA-DR+						
After 6 months of cART	.833	**.010**	.086	.771	.017	.966
After 12 months of cART	.900	**.037**	.345	.328	−.041	.905
ΔCD4 + CD38 + HLA-DR+						
After 6 months of cART	.167	.693	.530	.051	.250	.516
After 12 months of cART	.900	**.033**	.600	.067	.273	.446

P*: associations analyzed with Spearman’s test. **Bold** font if significant *p* value

In individuals with primary HIV infection, KTR at baseline was positively associated with the reduction in proportion of CD8 + CD38 + HLA-DR+ T cells after 6 (ρ .833, *p* = 0.010) and 12 months of cART (ρ.900, *p* = 0.037). Furthermore, in individuals with primary HIV infection a positive association was found between baseline KTR and reduction in proportion of CD4 + CD38 + HLA-DR+ T cells after 12 months of cART (ρ .900, *p* = 0.033) (Table 3). In late presenters and early presenters, no associations were found between baseline KTR and the reduction in proportion of CD8 + CD38 + HLA-DR+ and CD4 + CD38 + HLA-DR+ T cells after 6 and 12 months of follow-up (Table 3).

Negative associations between baseline KTR and CD4/CD8 ratio was found in early presenters at baseline, and after 6 and 12 months of follow-up (data not shown). No associations between KTR and CD4/CD8 ratio were found in individuals with primary HIV infection or late presenters at any time point.

No association was found between viral load and KTR at baseline in primary HIV infection and early presenters. In contrast, in late presenters viral load was positively associated with KTR at baseline (ρ .527, *p* = 0.033) (data not shown). Furthermore, at baseline no associations were found between viral load and proportion of CD8 + CD38 + HLA-DR+ and CD4 + CD38 + HLA-DR+ T cells in primary HIV infection, or early and late presenters.

Finally, no significant associations between KTR at baseline and immune recovery were found in any of the groups HIV-infected individuals during the follow-up period (data not shown).

## Discussion

The present study extends the knowledge of the impact of KTR in HIV infection by demonstrating that early initiation of cART is associated with lower KTR set point after 6 and 12 months of cART when compared to initiation of cART with CD4+ T cells counts less than 200 cells/μL. However, early initiation of cART did not result in normalization of KTR. Interestingly, in early presenters higher baseline KTR was found to be associated with higher immune activation at both baseline and after initiation of cART. Importantly, KTR was positively associated with a larger decrease in immune activation after 6 and 12 months of cART in patients with primary HIV infection.

Increased KTR has been described in HIV-infected individuals [6, 9, 16, 18, 30], and recent studies showed reduction in KTR after initiation of cART [6, 15–19]. It is worth noticing that among the previously conducted studies only Jenabian et al. included patients with early HIV infection (estimated time from infection <180 days) [15]. To our knowledge, our prospective study is the first to compare the effect of cART on KTR in HIV-infected patients with primary and chronic infection. At baseline, a lower KTR was found in individuals with primary HIV infection compared to late presenters, while primary HIV infection had higher KTR compared to early presenters. Interestingly, after 6 and 12 months of cART primary HIV infection and early presenters had comparable KTR set points, while late presenters had higher KTR set point than the two other groups. Importantly, all groups of HIV-infected individuals consistently had higher KTR than healthy controls during the entire study period. Several studies have described reduction in KTR after initiation of cART, without reaching the level found in healthy controls [17–19]. One study even found normalization of KTR after initiation of cART [15], but this finding has not been confirmed. Our data support and extend previous findings by demonstrating that early initiation of cART either during primary HIV infection or in early presenters has a beneficial effect on KTR set point compared to initiation of cART in late presenters.

Immune activation and chronic inflammation have been described as independent predictors of disease progression in HIV-infected individuals [31, 32]. Furthermore, immune activation and inflammation may be drivers of non-AIDS comorbidity and mortality in HIV-infected individuals [33]. Immune activation in HIV-infected individuals is commonly assessed by co-expression of activation markers CD38 and HLA-DR on T-cells [34, 35]. The proportion of CD4+ and CD8+ T cells co-expressing CD38+ and HLA-DR+ has been described to correlate with morbidity and mortality in HIV infected individuals [33, 36], and was therefore used to determine immune activation in this study. The association between KTR and immune activation in HIV infection has been described previously [15, 17, 22]. Thus, positive associations between KTR and neopterin [17] and between KTR and reduction in CD4/CD8 ratio [22] in chronic HIV-infected individuals have been reported. Furthermore, positive associations between KTR and CD8 + CD38 + HLA-DR+ T cells in untreated early HIV-infected patients have been described [15]. In order to more closely examine the relationship between immune activation and KTR in HIV-infected individuals, we investigated associations between KTR and CD8 + CD38 + HLA-DR+ and CD4 + CD38 + HLA-DR+ T cells. As expected, evidence of immune activation was found in all groups of HIV-infected individuals with the highest proportion of immune activated cells in individuals with primary HIV infection prior to initiation of cART. A strong positive association between KTR and proportion of CD8 + CD38 + HLA-DR+ T cells was found at baseline in both individuals with primary HIV infection and early presenters, in accordance with previous studies [15]. Interestingly, positive associations between baseline KTR and immune activation after 6 and 12 months of cART were found in early presenters.

Importantly, in individuals with primary HIV infection higher KTR at baseline was associated with greater reduction in proportion of CD8 + CD38 + HLA-DR+ T cells, both at 6 and 12 months of follow-up. Thus, a differential effect of KTR on immune activation was found in individuals with primary HIV infection, early presenters, and late presenters. During primary HIV infection interferon-γ (IFN- γ) levels steadily increase, with a peak approximately 3 weeks after the infection [37]. This may be associated with a beneficial immune response, which may help to control viral replication and to obtain a lower viral load set-point, and thus the level of immune activation during the chronic phases of the infection. IDO activity is highly enhanced by IFN- γ during inflammation [38]. We speculated that higher KTR during primary HIV infection is related to high IFN- γ activity, and may be linked to an initially beneficial host immune response, which may lead to a lower viral load and lower chronic immune activation in the chronic phases of infection. This beneficial association between KTR and immune activation may be lost with the establishment of an unregulated and deleterious chronic inflammation and immune activation during the chronic phases of HIV-infection, where IFN- γ levels decline to a steady state comparable to healthy controls [39]. Further studies in patients with primary HIV infection are warranted to elucidate this mechanism.

Little is known about the role of KTR as a predictor of immune recovery following cART. Data are scarce and contrasting. Thus, Byakwaga and colleagues described KTR before cART as a predictor of immune recovery [6], while Chen proposed opposite results [18]. Both these studies included only patients with chronic HIV infection. In our study, no correlation between baseline KTR and immune recovery was found in either primary or chronic HIV infection.

The main limitation in the present study is the number of individuals included and especially the low number of samples available at the follow-up. This may result in type II errors. Moreover, the cohort presented in the present study was not serocoincident. Therefore, comparing KTR among all the groups at the time of primary HIV infection was not possible. Thus, higher KTR in primary HIV infection compared to early presenters may be due to the fact that individuals who initiated cART during acute HIV sought prompt medical care because of more severe symptoms and, consequently, more immune activation. Furthermore, the study is descriptive, and conclusions on causality cannot be drawn. The small sample size prevented us from performing other statistical analysis, than descriptive analyses of the data. Finally, several comparisons were made without adjusting for multiple comparisons increasing the risk of type I errors. Thus, results should be interpreted taking the a priori hypothesis into account.

## Conclusions

In conclusion, initiation of cART during either primary or early HIV infection had a beneficial effect on KTR set point at follow-up compared to initiation of treatment in late presenting HIV infection. However, even in individuals with primary or early HIV infection KTR was only partly normalized. Interestingly, baseline KTR in treatment naïve patients presenting for care with primary or early HIV infection was positively associated with immune activation. Furthermore, individuals who initiated cART during primary HIV infection showed the largest reduction in immune activation, and decrease in immune activation was positively associated with baseline KTR. These findings suggest the importance of early cART in order to reduce KTR. Finally, an important role for KTR in immune activation is suggested.

## Abbreviations

cART: combined antiretroviral treatment
EP: Early presenters
IDO: Indoleamine 2,3-dioxygenase
KTR: Kynurenine tryptophan ratio
LP: Late presenters
PHI: Primary HIV infection
